# Transcriptomic profiling of cytomegalovirus infection in cardiac transplantation: proof-of-concept for a new strategy in tissue markers application

**DOI:** 10.3389/fimmu.2025.1581151

**Published:** 2025-05-16

**Authors:** Ilaria Barison, Diego Perazzolo, Chiara Castellani, Alessia Giarraputo, Elisabetta Rossi, Luca Vedovelli, Sonia Anna Minuzzo, Chiara Tessari, Nicola Pradegan, Giuseppe Toscano, Francesco Tona, Cristina Basso, Gino Gerosa, Susanna Mandruzzato, Davide Abate, Dario Gregori, Annalisa Angelini, Marny Fedrigo

**Affiliations:** ^1^ Cardiovascular Pathology, Department of Cardiac, Thoracic and Vascular Sciences and Public Health, University of Padova, Padova, Italy; ^2^ Department of Surgery, Oncology and Gastroenterology, Oncology Section, University of Padova, Padova, Italy; ^3^ Immunology and Molecular Oncology Diagnostics, Veneto Institute of Oncology (IOV) - Istituto di Ricovero e Cura a Carattere Scientifico, Padova, Italy; ^4^ Unit of Biostatistics, Epidemiology and Public Health, Department of Cardiac, Thoracic, Vascular Sciences, and Public Health, University of Padova, Padova, Italy; ^5^ Division of Cardiac Surgery, Department of Cardiac, Thoracic and Vascular Sciences and Public Health, University of Padova, Padova, Italy; ^6^ Cardiology Unit, Department of Cardiac, Thoracic, Vascular Sciences and Public Health, University of Padova, Padova, Italy; ^7^ Department of Molecular Medicine, University of Padova, Padova, Italy

**Keywords:** cytomegalovirus, heart transplant, rejection, transcriptomic profiling mRNA, miRNA, biomarkers

## Abstract

**Background:**

Cytomegalovirus (CMV) infection is a relevant threat to heart-transplanted patients during the first year after surgery, leading to increased morbidity and, in some cases, mortality. This *proof-of-concept* study aims to assess the transcriptomic profile of CMV infection in cardiac transplanted patients as a new diagnostic approach to discriminate infection and Acute Cellular Rejection (ACR) on EMB specimens.

**Methods:**

We performed a microarray-based messenger RNA (mRNA) and micro-RNA (miRNA) profiling. We analyzed three patient groups in the setting of CMV viremia and inflammatory infiltrate: a control group (n=5), an ACR group (n=5), and an infection group (n=6). Differentially expressed mRNA and miRNA were further investigated through bioinformatic pathway analysis.

**Results:**

Focusing on infection vs rejection comparison, we investigated the role of the 18 differentially expressed mRNAs and the 12 miRNAs with the most significative p-value (gene level fold change, FC <-2 or >2, p-value <0.05). Based on the bioinformatic analysis, we explored the regulatory effects of these miRNAs on the mRNA pathways independently identified in the same samples. The results showed that two genes, IL7R and GZMK (-38.63 and -3.15 FC, respectively), and two miRNAs, mir-93-5p and mir-345-5p (-2.63 and -2.18 FC, respectively), are differentially expressed in infection and can be exploited to differentiate CMV-positive from ACR-positive EMB specimens, reaching an AUC of 0.87 and an accuracy of 91% at cross-validation.

**Conclusions:**

We have identified a distinctive combined molecular profile of mRNAs and miRNAs for infection in post-cardiac transplant follow-up. Based on IL7R, GZMK, mir-93-5p, and mir-345-5p we suggest a novel possible workflow to distinguish infection, where those markers are downregulated, from rejection, where they are overexpressed, on EMB specimens. This analysis showed good accuracy and promising predictive performance. The future combined analysis of these genes and these miRNAs through user-friendly techniques, such as quantitative PCR, could reduce turn-around time and improve our diagnostic power for distinguishing CMV infection from ACR in EMB specimens.

## 1 Introduction

Cytomegalovirus (CMV) is the most clinically relevant post-transplant infectious agent. CMV is a member of the beta-Herpesviridae family that, in the normal population, latently infects 50-90% of individuals, but normally takes an asymptomatic course (1). Differently, in cardiac transplanted patients, CMV infection impacts morbidity and mortality (2, 3), and some studies showed that CMV infection is a risk factor for Antibody Mediated Rejection (AMR) and Cardiac Allograft Vasculopathy (CAV) (4–7).

The cardiac transplanted patients can be stratified according to the donor/recipient (D/R) serological status. The highest risk is associated with the mismatch between donor-positive and recipient-negative (D+/R-). Based on the approach chosen by each transplant center, these patients may undergo pre-emptive therapy (based on the antiviral administration for early asymptomatic CMV viremia detected by surveillance testing) or antiviral prophylaxis (2, 8, 9).

Routine EMB tissue exhamination represents a crucial procedure to determine CMV replication on the graft and thus intervene with a proper antiviral therapy and taper the immunosuppression regimen accordingly. However, the discrimination between Acute Cellular Rejection (ACR) and the inflammation process triggered by CMV infection itself is quite cryptic. In case of suspected CMV infection, both nucleic acid and immunohistochemical tests should be performed on EMB specimens, e.g. Immunohistochemical staining on Formalin Fixed Paraffine Embedded (FFPE) EMB slices and Polymerase Chain Reaction with the nucleic acid extracted from EMB tissue. Those tests are designed to detect viral components, such as the CMV genome or proteins, in tissue samples. Still, these tests are prone to false results due to CMV activation’s patchy nature in tissue, and the EMB procedure sampling errors.

Seeking novel biomarkers associated with rejection monitoring has been investigated over years in tissue and liquid biopsy (10–13). Transcriptomic studies opened new horizons on cardiac rejection allograft monitoring, showing that Rejection Associated Transcript analyses are similar through solid organ transplants and can differentiate among Cellular Rejection, Antibody-Mediated Rejection, and no-rejecting EMBs (11, 14, 15). Unfortunately, most of these studies focused mainly on inflammation but not on the infection. This limitation urges the need for new approaches that could help the pathologist in characterizing the allograft’s status, differentiating the inflammation itself from the virus induced inflammation pattern.

In this *proof-of-concept* study, we investigate the transcriptomic profile of CMV-positive patients at mRNA and miRNA levels to determine new candidate biomarkers to distinguish inflammatory infiltrate caused by infection from that due to rejection on FFPE EMB specimens. For this purpose, we analyzed the mRNA and miRNA profile of FFPE EMB through high-density Clariom S Affymetrix GeneChip arrays (Thermofisher Scientific, USA) platform. We demonstrated that combining different biomarkers can significantly improve CMV infection detection and discriminate it from ACR during EMB pathological assessment.

## 2 Materials and methods

All the details about the Study design, patient selection, EMBs histological evaluation, RNA extraction protocol, Microarray-based mRNA and miRNA analysis protocols, bioinformatics, and statistical analysis are provided in the Extended Methods in **Supplementary Materials**.

## 3 Results

### 3.1 Study population characteristics

The patients enrolled in this study were divided into three groups according to the ACR grade and the CMV viremia. Recipients’ and Donors’ characteristics are shown in **Table 1**. Among selected patients, 68.75% were male, and the mean age was 61. Ischemic Heart Disease was the most common diagnosis leading to transplant. These patients were homogenous for Circulating DSA at the time of EMB, lymphocyte count, hepatic functionality, and Ejection Fraction. The control group showed slightly worse renal functionality and a higher time interval between heart transplant and EMB.

**Table 1:** Study population characteristics.

Features	Control (n=5)	Rejection (n=5)	Infection (n=6)	t-test p-value Control vs Rejection	t-test p-value Control vs Infection	t-test p-value Rejection vs Infection
**Recipient Age (y)**	61 ± 5.33	61.6 ± 9.97	54.3 ± 19.69	0.91	0.45	0.46
**Recipient gender (male, %)**	60	60	100	1	0.17	0.17
**Ischemic Heart Disease (%)**	60	40	66.7	0.57	0.84	0.43
**Dilated Cardiomyopathy (%)**	20	40	66.7	0.57	0.84	0.43
**Arrhythmogenic Right Ventricular Cardiomyopathy (%)**	20	0	16.65	0.37	0.90	0.36
**Obstructive Cardiomyopathy (%)**	0	20	0	0.37	–	0.37
**Donor Age (y)**	51 ± 20.29	64 ± 6.67	43 ± 17.67	0.76	0.52	0.22
**Donor gender (male, %)**	80	60	33.3	0.75	0.87	0.88
**Cold Ischemia Time (m)**	184.2 ± 49.88	195.4 ± 52.04	189.83 ± 61.33	0.75	0.87	0.88
**Circulating DSA at the time of EMB (d)**	0	0	33.3	–	0.18	0.18
**Time between HTx and EMB (d)**	207 ± 113.90	62 ± 10.86	77 ± 79.00	0.046	0.067	0.668
**Neutrophils (10^9^/l)**	4.10 ± 2.12	4.16 ± 1.77	5.24 ± 3.37	0.96	0.51	0.52
**Lymphocytes (10^9^/l)**	0.76 ± 0.40	0.69 ± 0.21	0.99 ± 0.78	0.75	0.56	0.41
**P-AST (U/l)**	46.4 ± 52.92	23.6 ± 4.92	22.4 ± 15.48	0.39	0.37	0.87
**P-ALT (U/l)**	25.8 ± 20.59	21.8 ± 10.14	24.4 ± 27.07	0.71	0.51	0.37
**P-γGT (U/l)**	24.2 ± 9.67	58.6 ± 47.86	94 ± 67.50	0.22	0.08	0.38
**e-GFR (µmol/l)**	42 ± 15.76	75 ± 11.62	63.2 ± 41.23	0.007	0.33	0.56
**Creatinine (µmol/l)**	146.6 ± 30.37	86.4 ± 13.30	149.5 ± 133.77	0.0077	0.96	0.30
***Cyclosporine* (µg/l)**	135 ± 57.05	172.2 ± 36.25	167.83 ± 48.43	0.27	0.33	0.87
***Ejection Fraction (%)***	63	59	58.83	0.45	0.97	0.22

Age, sex, and the main biochemical parameters are reported as mean ± standard deviation. Y, Years; d, days; m, minutes; HTx, Heart Transplantation; eGFR, estimated Glomerular Filtration Rate.The bold style was used to highlight the features reported in the first column from the numerical values reported in the other cells of the table.

### 3.2 Microarray transcriptomic analysis of FFPE EMBs

We analyzed the transcriptomic profile of our samples through a microarray-based platform. The bioinformatic analysis compared the expression levels of detected mRNAs in the Controls, the Rejection group, and the Infection group.

Notably, 293 genes were differentially expressed between Control and Infection groups (82 up-regulated and 211 down-regulated), 407 genes between Control and Rejection groups (126 up-regulated and 281 down-regulated), and 18 genes between Infection and Rejection groups (10 up-regulated and 8 down-regulated) (p-value <0.05).

**Figure 1:**
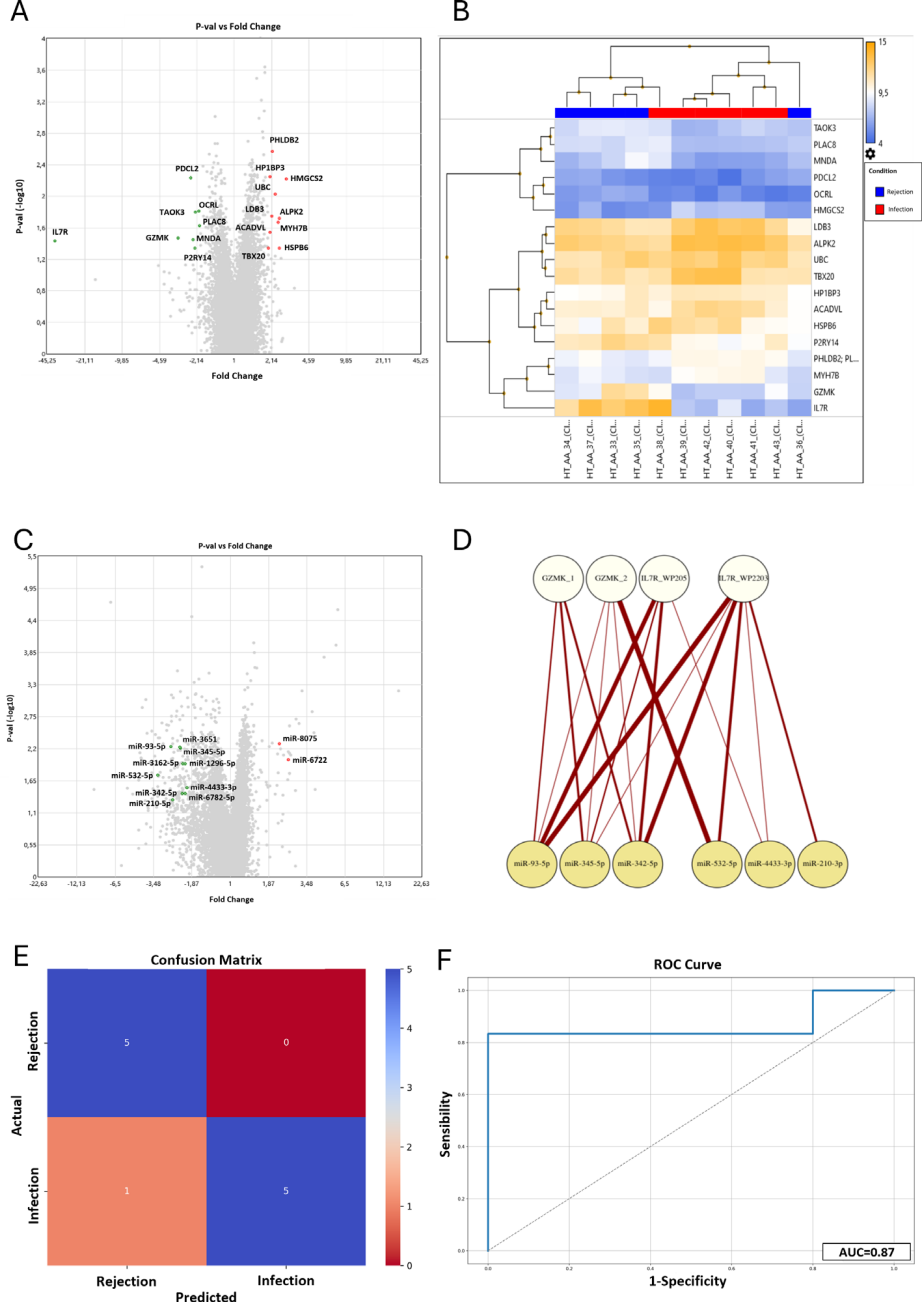
Results of the microarray mRNA analysis in the Infection vs Rejection group. **(A)** The Volcano Plot reported the differentially expressed genes between the Infection and Rejection groups. On the horizontal axis is reported the fold-change value (fold-change <-2 and >2), while the vertical axis reported the p-values (<0.05) of the same genes. **(B)** The Hierarchical Clustering reported the fluorescence level of each DEG (higher level in orange and lower level in blue). In the upper part of the graph is reported the clustering of the patients in the two study groups, Rejection in blue and Infection in red. **(C)** Results of the microarray miRNAs analysis in the Infection vs Rejection group. The Volcano Plot reported the differentially expressed miRNAs between the Infection and Rejection groups. On the horizontal axis is reported the fold-change value (fold-change <-2 and >2) while the vertical axis reports the p-values (<0.05) of the same miRNAs. **(D)** The bipartite graph between GZMK and IL7R pathways and miRNAs target genes, obtained by igraph library in R. The upper white nodes layer represents the Cytoscape pathways selected: GZMK glucose metabolic (GZMK_1), GZMK type I immune response (GZMK_2), IL7R WP205 - IL-7 signaling pathway (IL7R_WP205), and L7R WP2203 - Thymic stromal lymphopoietin signaling pathway (IL7R_WP2203). The lower yellow node layer represents the infection-related miRNAs. **(E)** Confusion Matrix. The panel shows the performance of the Support Vector Machine model (SVM) to classify infection and rejection at cross-validation. The y-axis represents the actual enrolled group (5 samples for rejection, and 6 samples for the infection group). The x-axis represents the samples classified by the SVM. **(F)** ROC curve analysis: Area Under the Curve (AUC) at cross-validation for infection and rejection discrimination is reported.

Since we aimed to define a possible molecular signature to distinguish infection from rejection at the tissue level, we focused our attention on the differentially expressed genes (DEG) that emerged in the comparison between these two study groups. In particular, PHLDB2, HP1BP3, HMGCS2, UBC, LDB3, ALPK2, MYH7B, ACADVL, HSPB6, and TBX20 resulted to be overexpressed in Infection compared to Rejection, while PDCL2, OCRL, TAOK3, PLAC8, GZMK, MNDA, IL7R, P2RY14 were downregulated in the same comparison (p<0.05) (**Figure 1A**). Afterward, we investigated the role of all these DEGs. We consulted several databases, such as the GeneCards database and WikiPathways TAC tool, focusing on their cellular functions and the biological processes they are involved in. This analysis revealed that these DEGs are mainly involved in cardiomyocyte metabolism (e.g. HMGCS2, ACADVL), IL-7 signaling and regulatory circuits of the STAT3 signaling (e.g. IL7R), DNA replication (e.g. HP1BP3) and cardiac progenitor differentiation pathways (e.g. PHLDB2, LDB3, ALPK2, TBX20) (**Supplementary Table S1**).

As shown in the Volcano Plot (**Figure 1A**), all the significantly differently expressed genes are quite homogeneous in terms of fold-change values, except for GZMK and IL7R. In detail, IL7R resulted downregulated in the Infection group compared to the Rejection (-38.63 fold-change; 12.69 (log2) Avg in the Rejection group vs 7.41 (log2) Avg in the Infection group). IL7R is highly expressed in conventional mature T-cells, except for regulatory T-cells that show a low level of IL7R on their surface (16, 17). Once activated, IL7R induces proliferative and anti-apoptotic signals mainly by activating several cellular pathways, such as JAK/STAT, PI3K/Akt, and MAPK/ERK pathways (18, 19). In addition, IL7R has been reported to activate chronic inflammation (20), autoimmunity (21), and allograft rejection in rodent models (22, 23). Likewise, GZMK is downregulated in the infection group (-3.15 fold-change; 9.27 (log2) Avg in Rejection group vs 7.62 (log2) Avg in Infection group). GZMK is a member of serine proteases that preferentially cleave after basic residues (24, 25), and it is expressed in different immune cell types, such as NK cells, cytotoxic T cells (26), and macrophages (27). GZMK is also reported to be involved in several pathologic conditions, such as cardiovascular diseases (28), vasculitis (29), and endothelial activation, promoting the release of proinflammatory cytokines from endothelial cells (30).

The heatmap reported in **Figure 1B** confirms that the DEG values are homogenous within each group, in terms of the expression level. Moreover, the hierarchical clustering correctly classifies nine out of eleven samples (82%), in agreement with histology and CMV serology. Only two samples were assigned to the wrong group: sample #36, selected as Infection but classified as Rejection by hierarchical clustering, and sample #38, selected as Rejection but classified as Infection by hierarchical clustering. In detail, the misclassification of these samples is linked to the values of GZMK and IL7R genes. In our cohort, the expression levels of these two genes were generally high in the Rejection group and low in the Infection group. Sample #36 showed a low level of expression of IL7R and GZMK genes, and the system classified it as “Infection” despite it being negative for the CMV serology test. On the other hand, sample #38 showed a high level of expression for IL7R and GZMK genes, and the system classified it as “Rejection” even though the CMV serology positivity. This misclassification suggests that those genes are crucial in differentiating Infections, with low expression, from Rejections, with high expression.

### 3.3 Microarray miRNAs analysis of FFPE EMB

The same total RNA extracted and analyzed for mRNA profiling was evaluated for miRNA profiling. The Clariom-S miRNA 4.0 chip tested the expression level of 2578 different miRNAs for each patient, and we performed the same comparisons conducted for the mRNA analysis (gene level fold-change <-2 or >2, p-value<0.05). The statistical evaluation of the raw data revealed that 513 miRNAs were differentially expressed between the Control and Rejection groups, with 477 miRNAs upregulated and 36 downregulated, while 111 (62 upregulated and 49 downregulated) between the Control and Infection groups. The comparison between the Infection and the Rejection groups showed that 386 miRNAs were differentially expressed (32 upregulated and 354 downregulated). Among these 386 miRNAs, 29 emerged differentially expressed only in the latter comparison. We further filtered out those results, focusing on the miRNA with the highest p-value. Hence we selected twelve hsa-miRNAs that showed the highest p-value in infection versus vs rejection comparison: miR-8075 (fold-change= 2.23; p-value= 0.0052), miR-93-5p (fold-change= -2.63; p-value= 0.0058), miR-3651 (fold-change= -2.26; p-value= 0.0059), miR-345-5p (fold-change= -2.25; p-value= 0.0061), miR-6722 (fold-change= 2.57; p-value= 0.0098), miR-1296-5p (fold-change= -2.08; p-value= 0.0112), miR-3162-5p (fold-change= -2.15; p-value= 0.0114); miR-532-5p (fold-change= -3.26; p-value= 0.0177), miR-4433-3p (fold-change= -2.02; p-value= 0.0293), miR-6782-5p (fold-change= -2.08; p-value= 0.0365), miR-342-5p (fold-change= -2.18; p-value=0.0371), and miR-210-3p (fold-change= -2.54; p-value= 0.0469) (**Figure 1C**).

Once determined our miRNAs of interest, we explored their function through a literature review. The screening of PubTator, GeneCards, and PubMed databases revealed that these miRNAs are mainly involved in PI3K/Akt, STAT, and HIF1α pathways, regulating cell proliferation and apoptosis, along with mitochondria stress response.

### 3.4 Analysis of mRNA and miRNA interactions in CMV infection

Once we identified the mRNAs and miRNAs differentially expressed in the Infection vs Rejection groups, we further investigated in silico the possible interactions among mRNAs and the target genes of miRNAs.

We consulted TAC and Microcosm databases to define the target genes of the selected miRNAs. Through a symmetric matrix (**Supplementary Figure S2**), we highlighted the number of shared genes among each couple of entries. In detail, it showed that only miR-93-5p targets some of the mRNAs identified as DEG in the Infection group.

Since we focused on the miRNAs shared target genes, we saw that the most relevant miRNAs were miR-93-5p, miR-345-5p, miR-532-5p, miR-342-5p, and miR-210-3p. Indeed, these miRNAs shared a higher number of target genes: miR-93-5p shared 101 target genes with miR-345-5p, 100 target genes with miR-532-5p, 78 target genes with miR-342-5p, and 66 target genes with miR-210-3p (**Supplementary Figure S2A**). Next, we represented these connections with an undirected weighted graph: the thickness of the edges is proportional to the number of targets shared between two miRNAs (**Supplementary Figure S2B**). Once again, the miRNAs that were more relevant were miR-93-5p, miR-345-5p, miR-532-5p, miR-342-5p, and miR-210-3p.

Interestingly, miR-93-5p targets some mRNAs identified in infection vs rejection: ACADVL, ALPK2, OCRL, PDCL2, and UBC. Using the EnrichR tool, we saw that these genes are mainly involved in fatty acid metabolism pathways (**Supplementary Figures S2C, D**).

### 3.5 The interplay among miRNA targets and GZMK and IL7R pathways

The analysis of mRNAs revealed that GZMK and IL7R are crucial for distinguishing infection from rejection. Thus, we investigated the interplay between the gene pathways and our selected miRNAs. Consulting the Cytoscape database, we chose the two most significant pathways for GZMK and IL7R based on the p-value associated. The genes reported to be involved in these pathways were matched with the miRNAs’ target genes to identify the shared ones, and the results are shown in (See **Supplementary Materials**, **Supplementary Table S2**). Notably, only seven miRNAs were shown to target some genes involved in GZMK and IL7R pathways, and their interplay is graphically represented by the bipartite graph reported in **Figure 1D**. Once again, miR-93-5p shared more target genes with the pathways analyzed, especially with IL7R pathways, such as MAPK1, JAK1, MYC, PIK3R2, STAT3, and CRLF2. MiR-345-5p and miR-342-5p target several genes in GZMK and IL7R pathways, such as ADHFE1, PTK2B, IRF1, PIK3R2, CRLF2, MAP2K2, MAPK3, LCK.

Thus, miR-93-5p and miR-345-5p showed the strongest connections with IL7R and GZMK pathways. Both of them were up-regulated in the Rejection group: mir-93-5p showed 5.98 Avg (log2) in the Rejection group vs 4.59 Avg (log2) in the Infection group; mir-342-5p showed 2.18 Avg (log2) in the Rejection group vs 1.6 Avg (log2) in the Infection group. Li et al. demonstrated that the miR-93 family takes part in PTEN regulation (31). PTEN is a tumor suppressor mutated in many cancers that antagonizes the PI3K/Akt pathway, modulating cell cycle progression and cell survival (32, 33). Furthermore, several studies demonstrated that this miRNA plays an important role in enhancing endothelial activities (34) and regulating integrin-β8 expression (35). Additionally, miR-345-5p showed several shared target genes with other miRNAs. This miRNA is reported to play a role in the regulation of HIF1α, and consequently, modulation of TGFβ/Smad2/Smad3 signaling (36), linked to ischemic damage response. Liu and colleagues reported that miR-345-5p can downregulate the TLR4/NF-κB pathway, altering inflammatory response and apoptosis (37).

Taken together, all these results showed that miR-93-5p and miR-345-5p are the best-ranked candidates to distinguish infection from rejection since they are down-regulated in CMV-positive patients and up-regulated in the Rejection group.

### 3.6 Performance assessment of the combined molecular markers

We are aware that our study population is small, and this leads to a reduced statistical power. This was confirmed by the *Post Hoc* Power analysis, which revealed that our cohort is associated with a value of (1-β) of 0.19, and, consequently, with a high risk of false negative results.

However, once defined our new panel of combined markers, we assessed the accuracy of this novel approach to distinguish infection from rejection. We implemented a classifier based on the identified markers using a Support Vector Machine with a polynomial kernel (KSVM). We then performed stratified cross-validation to evaluate its performance. As shown in the Confusion Matrix (**Figure 1E**), based on the level of expression of GZMK, IL7R, mir-93-5p, and mir-345-5p, the model correctly classified as “rejection” all the real rejection samples. On the other hand, the model correctly classified five out of six infection samples (83,3%), with only one misclassification. This performance is associated with an Infection Precision of 0.83, with an overall accuracy of 91% (F1 score of 0.91). Subsequently, the sensitivity and specificity analysis revealed that the combination of GZMK, IL7R, mir-93-5p, and mir-345-5p reached good results, with an Area Under the Curve (AUC) of 87% (**Figure 1F**).

In summary, these analyses showed that, although the power analysis highlighted a limited statistical power due to the small sample size, the model based on the combined marker panel exhibited a promising predictive performance.

## 4 Discussion

Heart-transplanted patients represent a fragile population. After surgery, they need to be strictly monitored to contrast possible rejection episodes. The primary defense against rejection is immunosuppressive therapy, which patients must take for their entire lives. Unfortunately, these treatments make them more susceptible to bacterial and viral infections. Cytomegalovirus is the most clinically relevant infection in cardiac transplanted patients during the first year of follow-up (1). CMV *de novo* infection or reactivation needs to be treated properly (2, 8). Thus, correct diagnosis is crucial to define the appropriate therapeutic protocol. With this study, we aimed to explore the role of mRNA and miRNA as new biomarkers to improve EMB interpretation and distinguish rejection from infection-related inflammatory infiltrate on graft tissue.

Current molecular tests to assess CMV infection on EMB specimens need improvement. Our study suggests that IL7R and GZMK are decisive in the discrimination between an activate inflammatory status, due to rejection and infection, and control stable patients. These genes were downregulated in the Infection group (-38.63 Fold-change for IL7R and -3.15 Fold-change for GZMK), compared to the Rejection group, suggesting an alteration in the regulatory mechanisms of their pathways. Many groups showed that IL7R (also called CD127) is downregulated in viral infection characterized by a latent phase (38–41). Interestingly, immune activation and CD4 lymphopenia have been associated with decreased IL7R expression (41). These results perfectly fit with the conditions observed in CMV-positive transplanted patients, who are exposed to a viral-induced stimulation in an impaired immunocompetence condition. Conversely, only a few and contrasting studies have been published about GZMK expression in viral infection (42). Of relevance, Verschoor and colleagues showed that GZMK expression decreased in some specific subsets of T cells in CMV-positive patients, and in older patients, who are known to be characterized by a fragile and altered immune response (43). Focusing on the connection between the pathways of GZMK and IL7R and the miRNA dysregulated in our comparison, we identified two key miRNAs that are up-regulated in the Rejection group: miR-93-5p (5.98 Avg (log2) in Rejection vs 4.59 Avg (log2) in Infection) and miR-345-5p (2.18 Avg (log2) in Rejection vs 1.6 Avg (log2) in Infection). These elements represent an important starting point for investigating new CMV diagnosis biomarkers. We could combine gene expression analysis (IL7R and GZMK) to assess the inflammatory status of the graft, and on the other hand, the assessment of miR-93-5p and miR-345-5p could be helpful to discriminate infection, where their levels of expression are decreased, from rejection. In clinical practice, the possible application of this novel and promising approach could be divided into two phases. First of all, thanks to IL7R and GZMK testing, we could perform a screening process and discriminate between stable and unstable patients, who show IL7R and GZMK increased expression levels compared to non-rejecting patients. Secondly, through mir-93-5p and mir-345-5p analysis, we could improve our capacity to distinguish infection, where those miRNAs show a lower level of expression, from rejection, where they are overexpressed. In this ideal new workflow, combining different biomarkers should be decisive in discriminating between infection and rejection. Indeed, inflammatory response is a dynamic process, and using a double level of investigation could improve our ability to identify infection-associated inflammatory infiltrate on tissue samples.

Over the past 30 years, multiple solid molecular and epidemiological evidences have connected CMV infection and the exacerbation of acute and chronic allograft rejection in solid organ transplants, especially heart transplants (4, 7, 44–47). The association between CMV and CAV, CAD, and vascular dysfunction has been deeply investigated at the molecular level, demonstrating the primary role of CMV in inducing focal inflammation, triggering atherosclerosis, and microvascular damage. These results were also confirmed in human hand transplantation and facial vascularized composite allotransplantation (48–50).

Additionally, this novel paradigm could be effective in improving CMV diagnosis and overcoming the limitations of molecular tests currently in use. Indeed, tests based on the detection of the viral genome are prone to false negative results, due to the focal reactivation of CMV and to sampling error of EMB. The application of PCR or immunohistochemistry analysis to detect the viral genomes or proteins may yield false-negative results when the EMBs fail to sample regions of focal CMV infection. Conversely, PCR may also produce false-positive findings by detecting viral particles present on tissue samples as a result of blood contamination rather than true myocardial viral localization. The analysis of genes that test the immune response to the virus could overcome these limits. Finally, thanks to this *proof-of-concept* study, we can optimistically foresee a wide clinical application of our promising results with cost-effective and user-friendly techniques, such as quantitative PCR (qPCR). Indeed, we identified four molecular candidate markers that could be easily analyzed on EMB specimen extract in all clinical laboratories, with no need for new platforms and minimal equipment costs.

Our study has some limitations. Unfortunately, our enrolled population is quite small, and this is due to the strict inclusion criteria applied in the selection phase. We aimed to evaluate the transcriptomic profile of the EMB in a setting of CMV infection without any other viral or bacterial coinfections, significantly reducing the number of eligible patients. Despite the modest population size, we obtained a huge amount of data thanks to the high-throughput transcriptomic platform employed, without any bias in transcript selection. Our cross-validation results demonstrated good performance, supporting the robustness of our findings despite the relatively low expected values. To address the current limitations, further validation is warranted in an independent cohort, ideally comprising a larger and more heterogeneous patient population. This will enhance the generalizability of our preliminary results to real-world clinical practice and help mitigate the risk of false negatives.

In conclusion, we explored the possibility of identifying new biomarker candidates in post-transplantation infection diagnosis. Our study is a *proof-of-concept* that infection and rejection are characterized by different transcriptomic profiles, both at mRNA and miRNA levels, shedding new light on the understanding of the pathophysiologic mechanisms that underlie these insidious conditions. Combining CMV-related gene analysis with rejection-related miRNA assessment could further improve our “resolution power” in distinguishing CMV infection from ACR, which can show an overlapping phenotype on histology. These novel and interesting results open up new possible innovative, and promising approaches for CMV infection diagnosis with the use of cost-effective qPCR applications, reduced turnaround time, and large-scale clinical applicability of our results.

## Data Availability

The microarray data have been deposited on Zenodo and they are available at this doi: 10.5281/zenodo.15295266. See the link: https://doi.org/10.5281/zenodo.15295266.
