# Supplementary material for: Transcriptomic profiling of cytomegalovirus infection in cardiac transplantation: proof-of-concept for a new strategy in tissue markers application
Source: Front Immunol. 2025 May 16;16:1581151. doi: 10.3389/fimmu.2025.1581151 (PMC12122309; doi:10.3389/fimmu.2025.1581151)
Supplement: Supplementary file 1 [file DataSheet1.docx]

Supplementary Material

# Extended Materials and Methods

## Study design and patients’ selection

# All patients enrolled underwent cardiac transplantation at the Cardio-Surgery Center Gallucci (Department of Cardiac-Thoracic-Vascular Sciences, and Public Health at the University Hospital of Padua, Italy), and they were monitored during all first year of follow-up post-transplant. Local monitoring protocol consists of scheduled blood tests and DSA dosage, FFPE EMB histopathological assessment, transthoracic or transoesophageal echocardiography, and general clinical evaluation during outpatient visits.

All participants were provided with complete information about the study, and all procedures performed were in accordance with the ethics standards of the institutional and/or national research committee and with the 2013 Helsinki Declaration and its later amendments or comparable ethical standards.

The local ethical committee approved the study protocol number 0062556.

This study selected 16 patients who underwent cardiac transplantation between January 2018 and December 2020. We excluded pediatric patients and adult patients who tested positive for coinfection. The EMBs were randomly selected within the first year of follow-up for patients who adhered to the selection criteria. The selected patients were divided into three study groups: a control group (n=5) that includes stable patients negative at EMB for rejection (ACR 0, pathological Antibody-Mediated Rejection, pAMR 0 ISHLT grade) and negative for CMV viremia; a rejection group (n=5) consisting of patients positive at EMB for ACR (≥3A-2R, pAMR 0 ISHLT grade) but negative for CMV viremia; and then an infection group that consists of six patients positive at EMB for ACR (≥1A-1R, pAMR 0 ISHLT grade) in the setting of CMV viremia (>1000 copies/mL).

## Histological evaluation of EMBs and assessment of cardiac allograft rejection

FFPE EMBs were routinely stained with hematoxylin-eosin. Immunohistochemistry was performed on tissue sections with the following antibodies: rabbit monoclonal anti-C4d (Biomedica Groupe, Vienna, Austria) and monoclonal anti-CD68 (Clone PG-M1; Dako Cytomation, Milano, Italy) using the immunoperoxidase method (1). Then, EMBs were carefully and concurrently reviewed by two expert pathologists, who reached an agreement on the grading. EMBs were classified according to the ISHLT 2013 updated international classification criteria for antibody-mediated rejection (2) and the ISHLT 2005 working formulation for acute cellular rejection (3) (Figure S1).

## Total RNA extraction from FFPE EMB tissue and microarray analysis for mRNA and miRNA

From each FFPE specimen, 20 slides of 10 μm thickness were cut and collected in a 2 ml microcentrifuge tube. The slices were dewaxed through three sequential incubations with xylol in agitation at 50°C for 10 minutes and centrifugated at 14000 rpm for 5 minutes. Once dewaxed, the sections were dehydrated with 100% ethanol and centrifugated for 5 minutes at 14000 rpm twice. Then the RNA was extracted using RecoverAll Total Nucleic Acid Isolation Kit (Life Technologies, USA) according to the manufacturer’s instructions. The amount of extracted RNA was assessed by Nanodrop One spectrophotometer (Thermo Scientific, USA).

## Microarray analysis of the mRNA and miRNA

Our study used high-density Clariom S Affymetrix GeneChip arrays (Thermofisher Scientific, USA), exploiting a single channel detection and short 25-mer probes. Concisely, 50 ng of total RNA were retrotranscribed into single-stranded cDNA (ss-cDNA) containing T7 promoter sequence at the 5’ end. The 3’ complementary cDNA strand was synthesized by adding an adaptor as a template, and a pre-in vitro transcription (pre-IVT) reaction was performed with 6 cycles of amplification, as suggested by the manufacturer's instructions. The double-stranded cDNA (ds-cDNA) obtained was used as a template for antisense RNA (cRNA) synthesis, and in vitro transcription (IVT) was performed overnight (14 hours) using T7 RNA polymerase. Then, 20 μg of cRNA were used as a template for ss-cDNA synthesis, and, after RNAse H digestion, it was purified using magnetic beads. Thus, 5.5 μg of ss-cDNA were fragmented by uracil DNA-glycosylase and apurinic/apyrimidinic endonuclease 1 and labeled with biotin using terminal deoxynucleotidyl transferase. Two hundred μl of this hybridization cocktail were loaded into the cartridge array Clariom-S 400-format and incubated for 16 hours in the Affymetrix GeneChip Hybridization Oven at 45°C and in rotation at 60 rpm. The arrays were then stained using the Affymetrix GeneChip Fluidics Station 450, according to the manufacturer’s FS450_0007 fluidics protocol, and scanned with the Affymetrix GeneChip Scanner. Finally, the GeneChipTM Command ConsoleTM converted the raw intensity signals in .CEL files.

We conducted the miRNA profiling on the same total RNA extracts by the Clariom-S miRNA 4.0 chips and FlashTagTM Biotin HSR RNA Labeling Kit (Thermofisher Scientific, USA). One hundred thirty ng of total RNA was diluted in a final volume of 8 μl, and it was added with Poly A Tailing Master. Then, the Poly(A) tailed RNA was Biotin-labeled 3DNATM, a technology that allows the signal to be amplified and increases the sensitivity of the analysis. A total of 21.5 μl were added to the Hybridization Mix, loaded into the GeneChipTM Cartridge Array, and eventually incubated for 16 hours in the Affymetrix GeneChip Hybridization Oven at 45°C and in rotation at 60 rpm. The staining and scanner phases followed the same protocols described above.

## Microarray Data Bioinformatic and Statistical Analysis

The .CEL files were imported and analyzed with Transcriptome Analysis Console (TAC) 4.0 (Applied Biosystems, USA). Differentially expressed transcripts and miRNAs were identified by Expression Gene Analysis, setting Fold Change >2 or <-2 and P-value <0.05 as filter criteria.

Then, a bioinformatic analysis of the TAC results was performed. The intersections between the mRNAs and the miRNAs datasets were defined through Multiple List Comparator, from Molbiotools, as already reported in literature (4).

Subsequently, using the R programming language and the related library “igraph”, we created an undirected weighted graph that shows the relationships between the target genes of miRNAs (5). Afterward, we built a pipeline that uses Cytoscape (6), an online tool, to identify the shared genes among the most relevant pathways that emerged from mRNAs analysis and the target genes of our miRNAs dataset.

Ultimately, through the R and igraph library, a two-layer bipartite graph (7) was built to connect our miRNAs set and the most relevant pathways defined.

The functions of all the genes, miRNAs, and pathways analyzed were better investigated with GeneCards, PubTator, PubMed, and WikiPathways databases.

Finally, we performed a Post Hoc power analysis and a cross-validation of our results. We estimated the statistical power of our results through G*Power software to assess the type II error in our small cohort of patients. Cross-validation analysis was conducted using the Python programming language, with the pandas and numpy libraries employed for data manipulation, and matplotlib for data visualization. Classification models were developed using the scikit-learn (sklearn) library. A polynomial kernel Support Vector Machine (SVM) model was used to classify infection and rejection cases based on the signal intensity values of GMK, IL7R, hsa-miR-93-5p, and hsa-miR-345-5p measured from microarrays. StratifiedKFold cross-validation was applied to ensure that class distribution was preserved in each fold. Model performance was evaluated using accuracy, precision, recall, F1-score, and ROC-AUC metrics.

References:

1. Fedrigo M, Feltrin G, Poli F, Chiara Frigo A, Benazzi E, Gambino A, et al. Intravascular macrophages in cardiac allograft biopsies for diagnosis of early and late antibody-mediated rejection. J Heart Lung Transplant. 2013;32(4):404–9.

2. Berry GJ, Burke MM, Andersen C, Bruneval P, Fedrigo M, Fishbein MC, et al. The 2013 International Society for Heart and Lung Transplantation Working Formulation for the standardization of nomenclature in the pathologic diagnosis of antibody-mediated rejection in heart transplantation. J Heart Lung Transplant. 2013;32(12):1147–62.

3. Stewart S, Winters GL, Fishbein MC, Tazelaar HD, Kobashigawa J, Abrams J, et al. Revision of the 1990 Working Formulation for the Standardization of Nomenclature in the Diagnosis of Heart Rejection. J Heart Lung Transplant. 2004;24(11):1710–20.

4. Perzanowska O, Smietanski M, Jemielity J, Kowalska J. Chemically Modified Poly (A) Analogs Targeting PABP : Structure Activity Relationship and Translation Inhibitory Properties. Chem- Eur J. 2022;28(42):e202201115.

5. Csárdi G, Tamás N. The igraph software package for complex network research. 2006.

6. Shannon P, Markiel A, Ozier O, Baliga NS, Wang JT, Ramage D, et al. Cytoscape : A Software Environment for Integrated Models of Biomolecular Interaction Networks. Genome Reasearch. 2003;13(11):2498–504.

7. Asratian AS, Denley TM, Haggkvist R. Bipartite graphs and their applications. Cambridge university press. 1998.

# Supplementary Figure 1


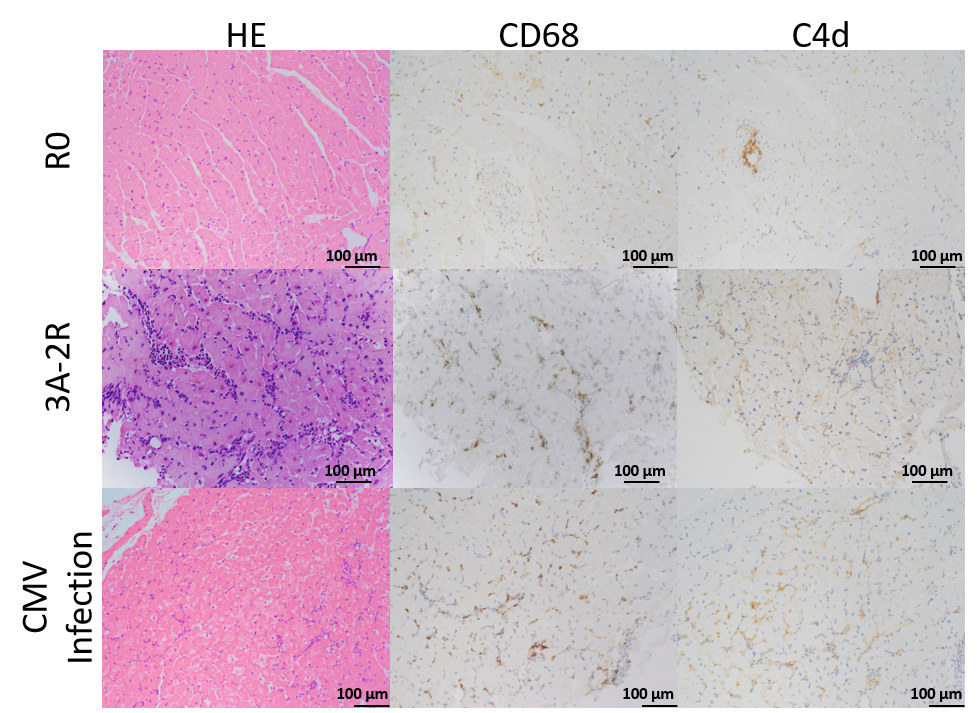


Figure S1: Histologic evaluation of the Endomyocardial Biopsies specimens with Haematoxylin-Eosin staining and anti-CD68 and anti-C4d immunohistochemistry in a control case (A), a rejection case (B), and infection (C). Scale bar = 100 μm. HE, Haematoxylin-Eosin; CMV, Cytomegalovirus; R0 – 3A-2R, ACR rejection score according to ISHLT guidelines.

# Supplementary Figure 2


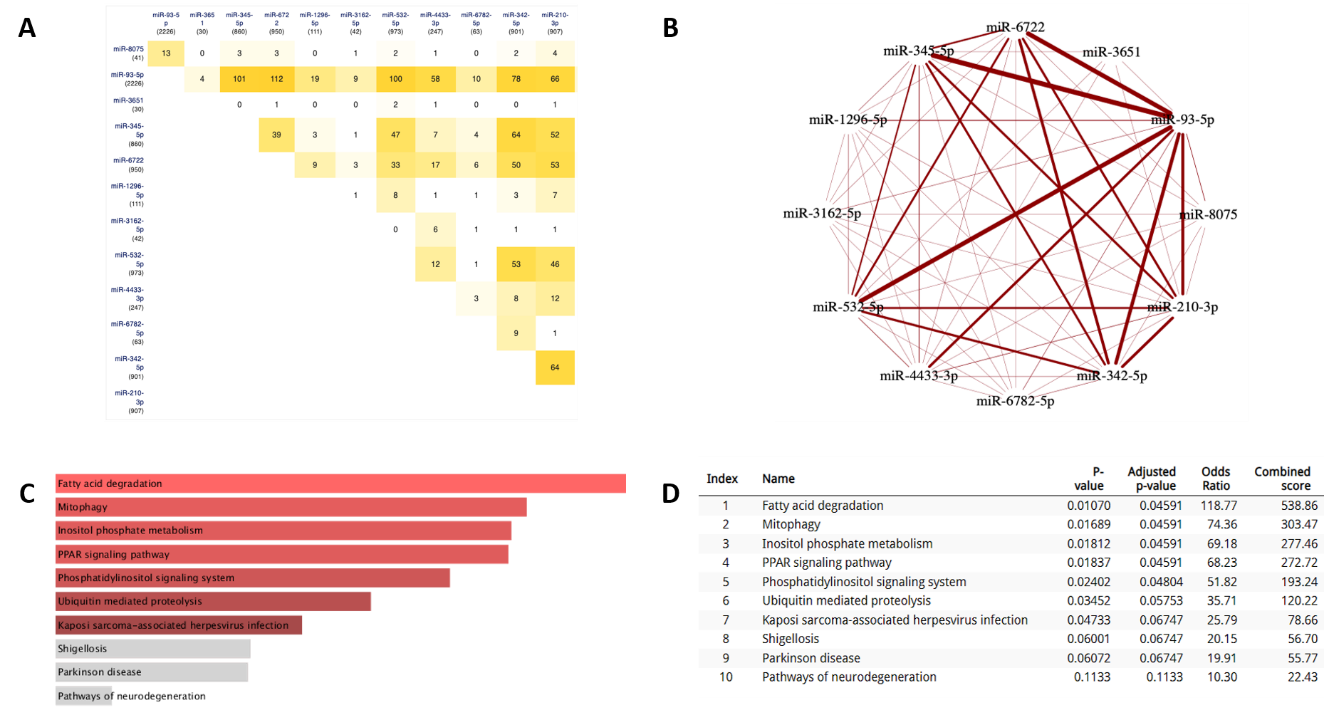


*Figure S2*. Analysis of the infection-related mRNAs and miRNAs connections. A) Symmetric matrix of miRNAs and mRNAs. The matrix matches the differentially expressed mRNA and the miRNA target genes that emerged in the infection vs rejection comparison. For each miRNA, the number of target genes is reported in brackets, while for the mRNA, the number of DEGs in Infection vs Rejection is reported. Each cell of the matrix is filled with the number of shared genes between the corresponding column and row. B) Undirected weighted graph of microRNAs. Graphical representation of the connections among miRNA target genes identified in the matrix. Each node of the graph represents a single miRNA, while the edge thickness is proportional to the number of common target genes. C-D) Pathways analysis of CMV infection signature genes regulated by miR-93-5p with EnrichR and KEGG 2021 Human database. Bar Graph of pathways in which the five genes targeted by miR-93-5p are reported, sorted by p-value ranking, in C. Pathways of the same miR-93-5p target genes, with p-value, adjusted p-value, Odds Ratio, and Combined Score are reported in D.

# Supplementary Table 1

Table S1. Summary of Differentially Expressed Genes (DEGs) between Infection and Rejection Groups. The microarray analysis of total RNA from the Infection and the Rejection groups showed 18 DEGs (reported in bold), 10 up-regulated, and 8 down-regulated. Their cellular functions, the biological processes they are involved in, their level of expression, and their p-value are reported.

| Gene Symbol | Cellular Function | Biological Process | Level of expression  Infection vs Rejection | p-value |
| --- | --- | --- | --- | --- |
| PHLDB2; PLCXD2 | Cadherin binding | Protein localization, cytoskeleton organization, negative regulation of wound healing, spreading of epidermal cells, regulation of epithelial to mesenchymal transition | Up-regulated | 0.003 |
| HP1BP3 | DNA and nucleosome binding | Cellular response to hypoxia, heterochromatin organization, nucleosome assembly, regulation of cell population proliferation, regulation of nucleus size, regulation of transcription, DNA-template | Up-regulated | 0.006 |
| PDCL2 | A putative modulator of heterotrimeric G proteins | - | Down-regulated | 0.006 |
| HMGCS2 | Catalyzes the first irreversible step in ketogenesis | Acetyl-CoA metabolic process, adipose tissue development, ketone body biosynthetic process, response to starvation | Up-regulated | 0.006 |
| UBC | Protease binding, protein tag, RNA binding, ubiquitin protein ligase binding | Modification-dependent protein catabolic process, protein ubiquitination | Up-regulated | 0.009 |
| OCRL | GTPase activator activity, phosphatase activity, small GTPase binding | Regulation of GTPase activity, signal transduction, inositol phosphate metabolic process | Down-regulated | 0.015 |
| TAOK3 | ATP binding, protein kinase inhibitor activity, transferase activity | Cellular response to DNA damage stimulus, DNA repair, regulation of MAPK cascade, protein phosphorylation | Down-regulated | 0.016 |
| LDB3 | Actin binding, cytoskeletal protein binding, metal ion binding, protein kinase C binding | Actin cytoskeleton organization, heart development, muscle structure development, sarcomere organization | Up-regulated | 0.018 |
| ALPK2 | ATP binding, protein serine kinase activity | Cardiac muscle cell development, epicardium morphogenesis, establishment of cell polarity, regulation of apoptotic process | Up-regulated | 0.019 |
| MYH7B | Actin filament binding, ATP binding, cytoskeletal motor activity | Muscle contraction | Up-regulated | 0.021 |
| PLAC8 | Predicted to enable chromatin binding activity | - | Down-regulated | 0.024 |
| ACADVL | Very-long-chain-acyl-CoA dehydrogenase activity, fatty-acyl-CoA binding, flavin adenine dinucleotide binding, identical protein binding | Fatty acid beta-oxidation using acyl-CoA dehydrogenase, epithelial cell differentiation, temperature homeostasis | Up-regulated | 0.028 |
| GZMK | Enables serine-type endopeptidase activity, enables protein binding, hydrolase activity | Cytolytic T lymphocytes and natural killer cell's immune response | Down-regulated | 0.034 |
| MNDA | Double-stranded DNA binding | B cell receptor signaling pathway, cellular response to DNA damage stimulus, positive regulation of apoptotic process | Down-regulated | 0.035 |
| IL7R | Antigen binding, cytokine receptor activity, interleukin-7 receptor activity | Negative regulation of T cell apoptotic process and T cell-mediated cytotoxicity, positive regulation of receptor signaling pathway via STAT | Down-regulated | 0.036 |
| HSPB6 | Chaperone binding, protein homodimerization activity, unfolded protein binding | Chaperone-mediated protein folding, positive regulation of angiogenesis | Up-regulated | 0.045 |
| TBX20 | DNA-binding transcription activator activity, RNA polymerase II-specific DNA-binding transcription factor binding | Cardiac muscle tissue morphogenesis, muscle contraction, positive regulation of cardiac muscle cell proliferation, vasculogenesis | Up-regulated | 0.045 |
| P2RY14 | G protein-coupled purinergic nucleotide receptor activity, G protein-coupled UDP receptor activity | G protein-coupled receptor signaling pathway | Down-regulated | 0.046 |

# Supplementary Table 2

Table S2. Common genes between selected miRNAs target genes and GZMK and IL7R pathways gene list. The genes present in more than one pathway and/or targets of more than one miRNA are reported in red, the genes shared by GZMK and IL7R pathways are underlined, and the genes reported involved only in one pathway are in blue.

| **miRNA** | **GZMK glucose metabolic pathway** | **GZMK type I immune response pathway** | **IL7R WP205 – IL7 signaling pathway** | **IL7R WP2203 – Thymic stromal lymphopoietin signaling pathway** |
| --- | --- | --- | --- | --- |
| miR-8075 | - | - | - | - |
| miR-93-5p | **ADHFE1**  **NTHL1** | **IRF1** | **MAPK1**  **JAK1**  **MYC**  **PIK3R2**  **STAT3**  **CCND1** | **MAPK1**  **CRLF2**  **JAK1**  **MYC**  **STAT3**  **IL8**  **MAPK9** |
| miR-3651 | - | - | - | - |
| miR-345-5p | **ADHFE1**  **PTK2B**  **ADH4** | **IRF1** | **PIK3R2**  **PTK2B** | **CRLF2** |
| miR-6722 | **WDTC1** | - | **MAPK1**  **JAK3** | **MAPK1**  **EIF4EBP1**  **STAT6** |
| miR-1296-5p | - | - | - | - |
| miR-3162-5p | - | - | - | - |
| miR-532-5p | - | **CASP12** | - | **EIF4EBP1**  **NFKB1**  **RPS6** |
| miR-4433-3p | - | - | **MAPK1** | **MAPK1** |
| miR-6782-5p | - | - | - | - |
| miR-342-5p | **ENO1**  **HMGA2**  **PFKM** | **CST7** | **MAP2K2**  **MAPK3**  **BCL2L1**  **IL2RG** | **LCK**  **MAP2K2**  **MAPK3**  **BTK**  **RELB**  **STAT4** |
| miR-210-3p | - | - | - | **LCK**  **LYN**  **STAT6** |
